# VpROM: a novel variational autoencoder-boosted reduced order model for the treatment of parametric dependencies in nonlinear systems

**DOI:** 10.1038/s41598-024-56118-x

**Published:** 2024-03-13

**Authors:** Thomas Simpson, Konstantinos Vlachas, Anthony Garland, Nikolaos Dervilis, Eleni Chatzi

**Affiliations:** 1 Department of Civil, Environmental, and Geomatic Engineering, ETH Zürich, Stefano-Franscini Platz 5, 8049 Zürich, Switzerland; 2 Sandia National Laboratories, Eubank Boulevard, Albuquerque, 87123 NM USA; 3 Dynamics Research Group, Department of Mechanical Engineering, University of Sheffield, Mappin Street, Sheffield, S1 3JD UK

**Keywords:** Parametric reduction, Reduced Order Models (ROMs), Conditional VAEs, Uncertainty, Civil engineering, Mechanical engineering

## Abstract

Reduced Order Models (ROMs) are of considerable importance in many areas of engineering in which computational time presents difficulties. Established approaches employ projection-based reduction, such as Proper Orthogonal Decomposition. The limitation of the linear nature of such operators is typically tackled via a library of local reduction subspaces, which requires the assembly of numerous local ROMs to address parametric dependencies. Our work attempts to define a more generalisable mapping between parametric inputs and reduced bases for the purpose of generative modeling. We propose the use of Variational Autoencoders (VAEs) in place of the typically utilised clustering or interpolation operations, for inferring the fundamental vectors, termed as modes, which approximate the manifold of the model response for any and each parametric input state. The derived ROM still relies on projection bases, built on the basis of full-order model simulations, thus retaining the imprinted physical connotation. However, it additionally exploits a matrix of coefficients that relates each local sample response and dynamics to the global phenomena across the parametric input domain. The VAE scheme is utilised for approximating these coefficients for any input state. This coupling leads to a high-precision low-order representation, which is particularly suited for problems where model dependencies or excitation traits cause the dynamic behavior to span multiple response regimes. Moreover, the probabilistic treatment of the VAE representation allows for uncertainty quantification on the reduction bases, which may then be propagated to the ROM response. The performance of the proposed approach is validated on an open-source simulation benchmark featuring hysteresis and multi-parametric dependencies, and on a large-scale wind turbine tower characterised by nonlinear material behavior and model uncertainty.

## Introduction

The use of Reduced Order Models (ROMs) in structural dynamics simulations forms a main ingredient of research revolving around the use of accelerated surrogates for the purpose of Structural Health Monitoring^1,2^, digital twinning^3,4^ and uncertainty quantification^5,6^. Reduced order modeling techniques are often categorised in terms of *purely data-driven* methods or *physics-based* methods. *Purely data-driven* methods employ input and output simulations, or even recorded data, of the system of interest to learn the underlying dynamics^7,8^. The advent of computing power, leading to increasingly deep machine learning architectures, has rendered such methods extremely capable of recreating even complex dynamics^9,10^. However, such methods always remain limited by the breadth and quality of the data used to train them^11,12^. *Physics-based* methods, on the other hand, allow for the creation of structured Reduced Order Model (ROM) representations, initiating from the equations of motion and projecting these onto a lower dimensional space upon which they can be solved^13–15^. Such a formulation maintains a stronger physics connotation and is, in this sense, often easier to interpret.

Reduction methods, that rely on the principle of projection and are capable of addressing nonlinear and/or parametric systems, often exploit Proper Orthogonal Decomposition(POD)^16^. These methods involve the execution of evaluations of the Full Order Model (FOM) and the use of the output response of these simulations, the so-called snapshots, for determining an appropriate reduction basis^13,17^. An alternative to POD methods is that of Proper Generalized Decomposition (PGD)^18^. Although, PGD is in someways inspired by POD methods, it has the very key difference of being an a-priori method, not requiring any simulation of the FOM in order to construct the ROM. These methods have also had significant success in various dynamical systems^19,20^.

A straightforward approach to POD-based Model Order Reduction (MOR) consists of constructing a global POD basis, in which data snapshots from simulations carried out at different points in the parameter/phase space are all stacked together. From this collection, a single (global) projection basis is then extracted. This method is widely used and has proven robust performance for a number of applications^21–23^, however, in the case of nonlinear systems, the POD only provides an optimal approximate *linear* manifold^24^. As such, with moderate to large nonlinearities, the respective POD-based reduction can become inefficient and requires the retention of several modes (at the cost of reduction), or even fails entirely^25^. Similarly, with parametric models, a global POD reduction basis can yield poor performance or become computationally inefficient^26^.

To this end, alternative strategies can be employed as a remedy, relying on either enriched low-order subspaces or a pool of local POD bases. The first technique is exemplified in^27,28^, where the authors make use of *enriched* reduction bases, in which underlying linear modal or vibration modes-based subspaces are enriched with modal derivatives in order to capture moderate geometric non-linearities. The second approach, on the other hand, relies on a library of pre-assembled, *local* ROMs, which are highly successful in approximating localised phenomena^29,30^. In this context, *local* ROMs can be defined with respect to time, implying the assembly of projection subspaces, and thus ROMs, which only capture the dynamics on a certain time window of the full behavior^31^. Thus, each ROM of the corresponding library refers to a different time window of the response, establishing locality. Alternatively, the local nature of these bases may refer to certain regions or subdomains of the input parameter space^32^, estimated through uniform^33,34^ or adaptive error estimators^35^, which perform a model or basis selection operation between the local ROMs during model evaluations. Such local bases can better deal with more heavily nonlinear and parameter-dependent systems, as they enable an indirect form of clustering of the parameter space (including time if needed), thus providing an accurate subspace approximation for the governing equations of motion at any parametric sample^36^.

Although this family of schemes can be considered an established pathway, when deriving an actionable ROM that serves across a broad range of operational conditions efficiency can be compromised and is highly sensitive to the basis selection technique from the assembled library of local ROMs. In this context, state of the art techniques employ clustering^37^ or interpolation^31^ operations performed in the proper manifold to maximise precision. On the other hand, recent contributions suggest that machine learning-inspired techniques can increase utility and improve performance of ROMs, whilst achieving an automated training process^38,39^. Inspired by the latter, in this work, we suggest substituting interpolation- or clustering-based schemes with an ML-based generative model, while retaining the projection-based reduction that guarantees domain-wide accuracy. Our approach aims to increase efficiency and robustness of the ROM by approximating the generalised mapping between parametric inputs and local projection bases, and the resulting ROMs, via the use of generative modeling.

Generative models are a group of statistical models that can serve for generating outputs from observed/simulated systems, under unseen initial conditions, loads, or properties outside those used in the original training set . This can be accomplished via conditioning on a parametric vector that reflects the characteristics of the system at hand. Formally, such a generative model learns the joint distribution $P(\varvec{X},\varvec{p})$ of the observed data $\varvec{X}$ and the parameter set $\varvec{p}$. As such, a generative model learns the distribution of the data itself hence allowing new samples to be drawn for simulation of new (previously unobserved or not simulated) outputs. More relevantly for our case, a generative model learns the distribution $P(\varvec{X}\vert \varvec{p})$, which is the distribution of the data given a certain parameter vector. The utility of such a generative model is twofold; via learning the joint distribution of reduction bases and associated model parameters, we can generate the local basis corresponding to any parameter sample, whilst further capturing the uncertainty on this inference.

One popular branch of generative models is deep generative models, which make use of deep neural networks as powerful and flexible nonlinear approximators that are suitable for modelling complex dependencies. The two most common examples of modern deep generative models are the GAN^40^ and the Variational AutoEncoder (VAE)^41^. Both architectures have garnered significant interest in a wide range of fields, ranging from the traditional machine learning subjects of computer vision^40^ and natural language processing^42,43^, to the domains of life sciences for novel molecule development^44,45^ and de-noising and analysis of Electron-microscopy images^46,47^. Recently, diffusion models have become the state of the art, beating the performance of GANs and VAEs in typical tasks for generative models such as image and video generation^48,49^. With regards to structural engineering, significant works utilising deep generative models include the application of GANs to nonlinear model analysis^50^ and the use of VAEs for wind turbine blade fatigue estimation^51^.

In this work, we tackle the problem of generating local bases at unseen parameter/input values, making use of a VAE as a nonlinear generative model. The VAE model was chosen due to its proven ability to learn highly nonlinear manifolds and efficiently work with high dimensional data. VAEs have the additional advantage of estimating uncertainty on the predicted bases. The VAE model was used to replace clustering or interpolation methods previously used for basis generation^31,37^ with the aim to improve accuracy, tackle high dimensional dependencies, and allow for uncertainty quantification. The structure of this paper is organised as follows: Section "Parametric reduced order modelling" gives a background on parameteric reduced order modelling and the current state of the art regarding projection methods for treating nonlinear parameteric systems. Section "VpROM: Coupling of Generative Models with projection-based ROMs" then describes the VAE model and how it is used in this work to replace the current state of the art interpolation or clustering methods in the parametric ROM. Section "Results" then demonstrates the use of the VAE-boosted parametric ROM on 2 example problems. Firstly on a three-dimensional shear frame, modeled with Bouc-Wen hysteretic nonlinearities in its joints, with multi-parametric behavior depending on system properties and excitation characteristics. Secondly, the method is demonstrated on a large scale Finite Element (FE) model of a wind turbine tower undergoing plastic deformation. In this case, the methodology is combined with a hyper-reduction scheme to demonstrate the full potential for reduction in computational time. In both cases, the VAE is shown to outperform the state of the art methods whilst also allowing for uncertainty in the ROM prediction to be captured. Section "Limitations and concluding remarks" concludes the paper by summarising the work and results achieved as well as the limitations of the method and by offering perspectives on future developments.

## Parametric reduced order modelling

The context of our work is the physics-based reduction of parameterised dynamical systems to derive an equivalent low-order surrogate of a FOM, namely FE formulations adopted for nonlinear structural dynamics simulations. In this context, projection-based reduction has been previously used for delivering response estimates^21^, as well as for parameter estimation^52^, or damage localisation and quantification tasks^53^.

This section first introduces the nonlinear equations of motion governing the problem at hand. Then, a projection-based reduction framework is described, along with the additional components needed for the treatment of parametric dependencies, largely following a similar methodology to the available state of the art approaches^31,54^. The efficiency considerations when propagating the dynamics in the low-order formulation are discussed last.

### Problem statement

We assume a general nonlinear dynamical system, characterised by the parameter vector $\varvec{p}=[p_1,...,p_{\textrm{z}}]^{\textrm{T}} \in \Omega \subset \varvec{R}^z$, which captures all system- and excitation-relevant parameters. Each realisation of $\varvec{p}$ corresponds to a unique configuration of the system at hand. Thus, the dynamic behavior of such a system is given by the following set of nonlinear governing equations of motion:

1 $$\begin{aligned} {\varvec{M}}\ddot{\varvec{u}}(t) + \varvec{g}\left( \varvec{u}(t), \dot{\varvec{u}}(t), \varvec{p}\right) = \varvec{F}(t,\varvec{p}), \end{aligned}$$

where $\varvec{u}(t) \in \varvec{R}^n$ represents the response of the system in terms of displacements, ${\varvec{M}} \in \varvec{R}^{n \times n}$ denotes the mass matrix, and $\varvec{F}(t, \varvec{p}) \in \varvec{R}^{n}$ the external excitation. The order of the system is expressed by the variable *n*, termed the full-order dimension, which physically represents the size of the coordinate space and thus the number of degrees of freedom in our system. Finally, the nonlinear effects are injected in the restoring force term $\varvec{g}\left( \varvec{u}(t), \dot{\varvec{u}}(t), \varvec{p}\right) \in \varvec{R}^{n}$. This term potentially encodes complex nonlinear phenomena of different nature, ranging from material nonlinearity to hysteresis or interface nonlinearities, which, in turn, depend on the parameter vector realisation and the response of the system.

### Projection-based model order reduction

In our work, we employ a Galerkin projection scheme, as described in^55^, to derive an efficient and accurate reduced order representation for the problem at hand, as described in subsection "Problem statement". We opt for a projection-based approach due to its interpretability and its utility for applications such as higher-level Structural Health Monitoring (SHM) systems^52^. Specifically, the derived ROM delivers a low-order, yet still physics-based, representation of the full physical space of the model. Thus, the ROM is not limited to capturing displacements but additionally infers stresses, strains, or any given physical field of interest at once, rather than deriving a ROM for specific elements or only at a few nodes^56,57^. The derivation approach of the parametric ROM is described in what follows in a step-wise manner. The approach assumes the availability of a high fidelity FOM, in our case a FE model that spatially discretises the full-order representation of the system in Eq. (1). Typically, a projection-based ROM relies on the premise that the dynamic response, in the present case the solution of Eq. (1), lies in a low-order subspace of size *r*, where *r* is orders of magnitude smaller than the FOM dimension, denoted by *n* ($r \ll n$). Thus, the following approximation holds:

2 $$\begin{aligned} {\varvec{u}\left( t\right) } \approx \varvec{V}\left( \varvec{p}\right) \varvec{q}{\left( t \right) } \end{aligned}$$

where $\varvec{V}\in \mathbb {R}^{n \times r}$ represents the ROM basis that expresses the aforementioned subspace and $\varvec{q}\in \mathbb {R}^{r}$ is the respective low-order coordinate vector. By substituting $\varvec{u}$ into Eq. (1) and pre-multiplying with $\varvec{V}^{T}$, thus performing a Galerkin projection, the following equivalent system is derived:

3 $$\begin{aligned} {{\tilde{{\varvec{M}}}} \ddot{\varvec{q}}\left( t \right) + {\tilde{\varvec{g}}}\left( {\ddot{\varvec{q}}}(t),{\dot{\varvec{q}}}(t), \varvec{p}\right) } = \tilde{\varvec{F}}\left( \varvec{p}, t \right) \end{aligned}$$

where $\tilde{\varvec{M}}=\varvec{V}^T\varvec{M}\varvec{V}$, $\tilde{\varvec{g}}=\varvec{V}^T\varvec{g}$ and $\tilde{\varvec{F}}=\varvec{V}^T \varvec{F}$. Key to a reduction that achieves an accurate low-order representation is the assembly of the projection basis $\varvec{V}$. Following the suggestions in^31^, we employ the POD technique.

Essentially, a set of orthonormal basis vectors is utilized to project the system of Eq. (1) onto a low dimensional subspace, as in Eq. (3). To achieve this, this strategy evaluates the full order FE model, summarised in Eq. (1), under realisations of a training set of parameters and gathers displacement samples to form the following matrix:

4 $$\begin{aligned} \hat{\varvec{S}} = \left[ \begin{array}{c c c c} \hat{\varvec{U}}\left( \varvec{p}_1 \right)&\hat{\varvec{U}}\left( \varvec{p}_2 \right)&\ldots&\hat{\varvec{U}}\left( \varvec{p}_{N_s} \right) \end{array} \right] \end{aligned}$$

where $\hat{\varvec{S}} \in \mathbb {R}^{n \times (N_t \times N_s)}$ is termed as the snapshot matrix, and $\hat{\varvec{U}}\left( \varvec{p}_i \right) \in \mathbb {R}^{n \times N_t}$ contains the time history of the response for every degree of freedom (DOF) for a given parametric realisation, henceforth termed as a snapshot. $N_t$ designates the number of simulation time steps, $\varvec{p}_i$ is the parametric input for snapshot *i* and $N_s$ is the number of snapshots. In turn, we search for a subspace $\varvec{V}\subset \mathbb {R}^{n}$ in which the displacement samples can be described in an optimal sense. It has been proven in^58^ that $\varvec{V}\in \mathbb {R}^{n\times r}$ can be obtained by properly truncating the set of left orthonormal vectors $\varvec{L}\in \mathbb {R}^{n \times n}$ that can be obtained via applying a Singular Value Decomposition (SVD) to the snapshot matrix $\hat{\varvec{S}}$. It has also been demonstrated that the projection error in Eq. (2) scales with the sum of the squares of the neglected singular values $\sigma$ due to truncation. Following the suggestions in^37^, a heuristic criterion is used to select the truncation order *r*:

5 $$\begin{aligned} \frac{\sum \limits _{i=r+1}^{n} \sigma _i^2}{\sum \limits _{i=1}^{n}\sigma _i^2} \le \epsilon \end{aligned}$$

where $\epsilon \in [0, 1]$ denotes a user-defined sensitivity parameter. As $\epsilon$ tends to zero, the truncation order increases, and the number of basis vectors $\varvec{L}_i$, which are considered, also increases, thus yielding a more accurate reduced representation. At the same time, the singular values might exhibit an initial fast decay rate, followed by a considerably slower one, as demonstrated in^55^. Thus, by setting $\epsilon =1e^{-4}$, for example, and examining the decay rate of the singular values $\sigma$, a proper reduced order *r* can be selected.

### MACpROM: treatment of parametric dependencies via clustering

As indicated in Eq. (1), the dynamic behavior of the system depends on a set of parameters that express system properties or traits of the induced excitation. Thus, the resulting response is strongly dependent on the parameter vector realisation and may be dominated by localised effects (in the parametric space) due to the corresponding activation of nonlinear terms. To address this, in our previous works in^59,60^, we have successfully employed a strategy relying on local POD bases, partially following the suggestions in^63^ and exploiting a cosine similarity measure, also referred to as the Modal Assurance Criterion (MAC) in the SHM domain^61^ as a clustering measure.

**Table 1:**
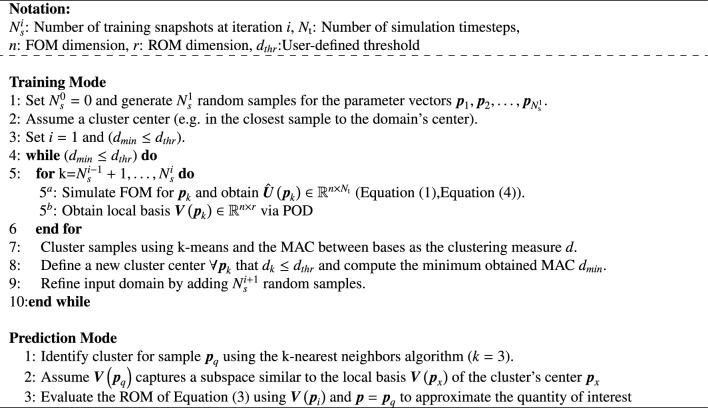
The algorithmic MACpROM framework. The maximum number of clusters can be used instead of the user-defined threshold $d_{thr}$ as a stopping criterion.

Specifically, the locality on the POD bases refers to forming clusters within the original parametric domain. In turn, in prediction mode, the framework assigns any validation sample to a cluster and uses the dedicated POD basis of the cluster (the cluster’s center) to reproduce the underlying FOM behavior accurately. However, since the dynamic behavior in each sample *i* is dominated by localized nonlinear effects captured by $\varvec{V}_i$, clustering is not performed directly on the parametric samples nor employs the usual distance metrics. Instead, the proposed technique clusters the retained modes in $\varvec{V}$, resulting after SVD and truncation for each FOM evaluation. The MAC is utilized as a comparative measure between projection modes of different parametric samples, evaluating the modes’ ability to capture similar nonlinear effects. Assuming $\varvec{w}_r$ and $\varvec{w}_s$ correspond to the $i_{th}$ truncated mode for the projection bases $\varvec{V}_r(\varvec{p}_r)$ and $\varvec{V}_s(\varvec{p}_s)$ respectively, the mathematical expression for the MAC reads:

6 $$\begin{aligned} \textrm{MAC}({\varvec{w}_r},{\varvec{w}_s})=\frac{|{\varvec{w}_r}^{T}{\varvec{w}_s}|^{2}}{({\varvec{w}_r}^{T}{\varvec{w}_r})({\varvec{w}_s}^{T}{\varvec{w}_s})} \end{aligned}$$

This measure is utilized to infer the value of new information that each mode captures. When summed over all modes of two bases, it helps to orientate the greedy-like sampling and the clustering during the training phase as explained in Table 1, where the final number of clusters is decided based on the choice of $d_{thr}$. A threshold value of $d_{thr}=0.90*r$ is used as a stopping criterion, where *r* is the number of retained basis vectors in $\varvec{V}$; that is the reduced order in Eq. (3). Alternative measures, such as the relative change to $d_{thr}$ between sampling events or the maximum number of clusters, can also detect convergence.

This approach is termed here as the Model Assurance Criterion parametric ROM (MACpROM) for reference purposes, and its respective algorithmic framework is summarised in Table 1. The elements of this approach have been validated in previous works and have been shown to be capable of delivering an accurate and efficient reduced-order representation of nonlinear systems^60,62^. In this work, this approach serves as an established reference scenario for the validation of the proposed VAE-boosted ROM, termed *VpROM*.

### CpROM: treatment of parametric dependencies via local basis coefficients interpolation

An alternative formulation for treating parametric dependencies in the context of nonlinear MOR has been proposed and verified with respect to state of the art approaches in^55^. Specifically, the authors proposed an interpolation approach on the local projection bases relying on the established techniques in^31^. To this end, a two-stage projection was introduced, thus allowing the dependence on parameters $\varvec{p}$ to be formulated on a separate level from that of the snapshot procedure or the local subspaces. Thus, after constructing a pool of local bases, each local basis $\varvec{V}_i$ is projected to the assembled global POD basis of the additionally projected to the global POD basis of the domain $\varvec{V}_{global}$ through a coefficient matrix $\varvec{X}_i$ as follows:

7 $$\begin{aligned} \varvec{V}_i = \varvec{V}_{global} \varvec{X}_i \end{aligned}$$

where $\varvec{V}_i \in \mathbb {R}^{n \times r}$, $\varvec{V}_{global} \in \mathbb {R}^{n \times \tilde{r}}$ and $\varvec{X}_i \in \mathbb {R}^{\tilde{r} \times r}$. The variable $\tilde{r}$ signifies the number of modes retained on the global basis $\varvec{V}_{global}$. In this manner, interpolation can be performed on the level of coefficient matrices $\varvec{X}_i$, which comprise a reduced dimension ($\tilde{r} \ll n$), thus removing any dependency on the large FOM dimension *n* and rendering the required operations more efficient. In turn, the respective matrices $\varvec{X}_i$ are projected and interpolated in an element-wise manner on the tangent space of the proper Grassmannian manifold and projected back on the original space to obtain the respective local ROM basis $\varvec{V}$ for any validation sample. This strategy is required for the local bases to retain certain orthogonality and positive-definiteness properties and is described in detail in^25,26^. A schematic visualisation of the approach, along with its algorithmic framework can be found in^55^. In our work, this framework is termed CpROM, adopting the same acronym as in the original work for reference purposes. This serves as an additional comparison ROM framework to validate the performance of the proposed *VpROM*.

### Hyper-reduction

Both the MACpROM and the CpROM frameworks, which were previously presented, are employed in conjunction with an additional operation, known as hyper-reduction, in order to achieve a substantial reduction in computational cost when dealing with nonlinear systems. Hyper-reduction refers to a second-tier approximation strategy, that addresses the bottleneck of updating and reconstructing the ROM system matrices due to the presence of the nonlinear terms in an online manner^63^. In essence, this technique relies on a weighted evaluation of the corresponding projections of the nonlinear terms only at a subset of the total elements in the spatial discretisation, thus providing substantially accelerated model evaluations. The detailed description of the method and the discussion of the existing alternative approaches are already covered in previous works and, thus, lie beyond the scope of this paper. The interested reader can refer to^64–66^. The validation case studies in our work make use of the Energy Conserving Mesh Sampling and Weighting (ECSW) technique presented in^36,67^.

## VpROM: Coupling of Generative Models with projection-based ROMs

Current state of the art methods for the creation of low-order surrogates of parameterised nonlinear dynamical systems rely on the use of interpolation or clustering methods for the estimation of local bases for given parametric configurations. This work introduces a nonlinear generative model, exploiting a conditional Variational AutoEncoder (cVAE) formulation, in place of these methods, with the aim of improving robustness and performance issues of the ROM by allowing for nonlinearities in the parameter-basis relation to be captured and for high dimensional dependencies to be better-dealt with. Furthermore, the derived *VpROM* allows for increased utility with regard to the capturing of uncertainty in the predicted bases.

### Variational autoencoder (VAE)

**Figure 1:**
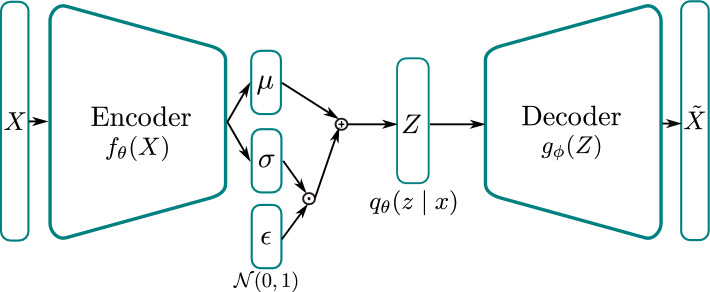
Architecture of a variational autoencoder (VAE).

The VAE first described in^41^ is a latent variable model, that is, a model in which it is assumed that the observations are driven by certain unobserved *latent* variables. Such latent variable models are popular in many areas of science and engineering and are often used to reduce the effective dimensionality of data since the dimension of the latent variables is reduced compared to that of the observations^68^. Indeed, such a concept is inherent to structural dynamics, as modal analysis, and its nonlinear extensions, all utilise lower dimensional representations to simplify the required analysis. In a probabilistic sense, modes can be considered latent variables, which are unobserved and drive the observed dynamics of the system. The VAE architecture as shown in Fig. 1 thus serves to infer relationships between the latent variables and the observed variables by means of deep neural network functions.

In the context of MOR, a VAE can be considered as a Bayesian implementation of a deterministic autoencoder; a popular deterministic deep learning technique, which has been often exploited for dimensionality reduction. Lee and Carlberg^56^ make use of a convolutional autoencoder, in conjunction with the nonlinear Galerkin method to construct ROMs for advection-dominated problems dynamics problems. Further work has combined an autoencoder with statistical regression methods to create fully data-based ROMs of nonlinear dynamical systems for structural dynamics^57,69^. A similar methodology known as Learning Effective Dynamics has also been shown to be effective in creating ROMs of some more classical nonlinear dynamical systems in various scientific disciplines^70,71^.

In a VAE model it is assumed that the data $\varvec{X}$ are characterised by a probabilistic distribution $p(\varvec{X})$, which we would like to approximate by means of a parameterised, and possibly simplified, distribution $p_{\phi }(\varvec{X})$ which is parameterised by the vector $\phi$. We assume that the complex distribution of the data is driven by a lower dimensional and more simply distributed hidden variable set $\varvec{Z}$, with assumed prior distribution $p(\varvec{Z})$. The concept here is that given a sufficiently powerful and flexible approximator, it is possible to learn a function that maps the simply distributed latent variables $\varvec{Z}$ to the complexly distributed data $\varvec{X}$ by learning the distribution $p_{\phi }(\varvec{X}\vert \varvec{Z})$^72^. This approximated distribution is found in the form of a deep neural network, namely the *decoder network*. This network is then parameterised by $\phi$ corresponding to the weights and biases. This results in the following expression for the generative model:

8 $$\begin{aligned} p_{\phi }(\varvec{X}) = \int {p_{\phi }(\varvec{X}\vert \varvec{Z})p(\varvec{Z})dz} \end{aligned}$$

The training of such a generative model necessitates the inference of those *decoder* parameters, $\phi$, that maximise the likelihood of the observations. It is here noted that the term observations in this case refers to synthetically generated data from FOM snapshots. This can be expressed as:

9 $$\begin{aligned} \phi = {\text {*}}{argmax}_\phi \prod _{i=1}^{N}\int {p_{\phi }(\varvec{X}^i\vert \varvec{Z})p(\varvec{Z})dz} \end{aligned}$$

The evaluation of this integral, however, presents a problem, as it is generally analytically intractable and computationally inefficient to approximate via sampling. For this reason, a second *encoder* network is introduced in the typical VAE setup, which is additionally parameterised by $\theta$. This allows the intractable posterior $p(\varvec{Z}\vert \varvec{X})$ to be approximated by the parameterised distribution $q_{\theta }(\varvec{Z}\vert \varvec{X})$ and hence creates a mapping from the observation space to the latent space. A variational approximation of the distribution of the latent space variable is also made whereby it is assumed that the latent variable takes on a certain known distribution $p(\varvec{Z})$. This variational approximation of the true posterior results in the following lower bound on the log-likelihood:

10 $$\begin{aligned} \mathcal {L}(\theta ,\phi ,\varvec{X})={{\,\mathrm{\mathbb {E}}\,}}_{q_{\theta }(\varvec{Z}\vert \varvec{X})}[log(p_{\phi }(\varvec{X}\vert \varvec{Z})]-D_{KL}(q_{\theta }(\varvec{Z}\vert \varvec{X})\vert \vert p(\varvec{Z}))) \end{aligned}$$

where $D_{KL}$ denotes the Kullback-Leibler divergence, a metric used to measure the similarity of distributions.

It is then this lower bound, known as the evidence based lower bound (ELBO), which is optimised with respect to the parameters of the two networks, $\theta$ and $\phi$. The maximisation of this function aims to i) improve the expected reconstruction loss of the decoder, i.e., the success in recovering observations from the latent variables, and ii) to minimise the KL divergence (and thus maximize the similarity) between the true and approximate posterior of the latent space. Once the VAE is trained based on this process, it is then possible to sample the latent space, using the inferred variational distribution, $q_{\theta }(\varvec{Z}\vert \varvec{X})$, and subsequently employ the decoder in order to recreate desired quantities of interest (outputs).

To optimise Eq. (10), it is necessary to estimate the gradients of the ELBO. Kingma et al.^41^ achieved this by using the *re-parameterisation trick*. By choosing the form of the approximate posterior to be a diagonal Gaussian parameterised by the encoder network, sampling from this distribution can be re-parameterised as follows;

11 $$\begin{aligned} \eta&= \mathcal {N}(0,I)\end{aligned}$$

12 $$\begin{aligned} q_{\theta }(\varvec{Z}\vert \varvec{X})&= \mathcal {N}(\varvec{Z}:\mu _{\theta }(\varvec{X}),\sigma _{\theta }(\varvec{X}))\end{aligned}$$

13 $$\begin{aligned}&=\mu _{\theta }(\varvec{X})+\eta \odot \sigma _{\theta }(\varvec{X}) \end{aligned}$$

in which $\eta$ represents a sample from the diagonal Gaussian distribution $\mathcal {N}(0,I)$ and $\mu _{\theta }(\varvec{X}),\sigma _{\theta }(\varvec{X})$ are the mean and standard deviation values of the latent space as output by the encoder network. The approximate posterior is then reformulated as being a stochastic draw $\eta$ and the deterministic mean and standard deviation values are predicted by the encoder.

This allows for evaluation of the expectation through sampling from a standard multivariate Gaussian, whilst the gradient can be assessed deterministically for each sample allowing the use of back propagation for training. Further, with the choice of a spherical unit Gaussian prior for $p(\varvec{Z})$, the KL divergence term can be calculated analytically^41^. This results in the following per sample, differentiable cost function, in which the expectation is evaluated using $N_v$ samples from the latent space per data point.

14 $$\begin{aligned} \mathcal {L}(\theta ,\phi ,x^i)=\frac{1}{2}\sum ^J_{j=1}(1+log((\sigma _j^{(i)})^2)-(\mu _j^{(i)})^2- (\sigma _j^{(i)})^2)+\frac{1}{L}\sum _{l=1}^{N_v}log(p_{\phi }(X^i,Z^{i,l}) \end{aligned}$$

where *J* denotes the dimension of the latent space and $Z^{i,j} =\mu _{\theta }(X^i)+\eta ^l\odot \sigma _{\theta }(X^i)$. The number of samples to take from the latent space to evaluate the expectation can even be 1 as in the original formulation of Kingma et al.^41^.

As mentioned above, in the VAE model the encoder and decoder functions are approximated using deep neural networks (DNNs). DNNs are a very widely used class of models that exploit multiple neural network layers applied one after another in order to approximate very complex functions more efficiently than shallow networks^73^. A thorough description of DNNs and their training can be found in^40^, in the work herein the DNNs utilised only made use of fully connected layers. In fully connected layers, the transform performed by each layer consists of the matrix multiplication of the input vector with a trainable weights matrix and the addition of a trainable bias vector. A nonlinear activation function, often a *tanh* or *sigmoid* is then applied element-wise to the output of this operation. This results in a very flexible and powerful model for learning general nonlinear relations^40^.

### VpROM: a conditional variational autoencoder (cVAE)-boosted ROM

**Figure 2:**
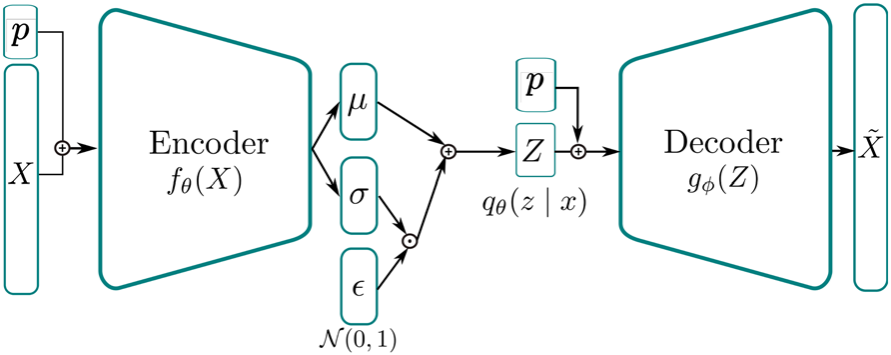
Architecture of a cVAE in which the conditioning variables are injected via concatenation with both the input vector $\varvec{X}$ and the latent vector $\varvec{Z}$. The input refers to the ROM basis coefficients $\varvec{X}$ in Eq. (7).

In our use case, we don’t simply wish to sample possible, plausible bases for our system of interest, rather, we want to sample these bases *conditioned* on given system and load (excitation) parameters. We can achieve this relatively straightforwardly by concatenating the conditioning parameters $\varvec{p}$ with the inputs of the VAE $\varvec{X}$, and with the latent space variables $\varvec{Z}$ as demonstrated in Fig. 2. Mathematically, the distribution approximated by the encoder now becomes $q_{\theta }(\varvec{Z}\vert \varvec{X},\varvec{p})$ and the distribution approximated by the decoder becomes $p_{\phi }(\varvec{X}\vert \varvec{Z},\varvec{p})$. To clarify the role of cVAE in the derived *VpROM*, the input referred to in Fig. 2 corresponds to the ROM basis coefficients $\varvec{X}$ in Eq. (7). Thus, Fig. 2 serves as a visualisation of the mapping process that the cVAE carries out to relate the parametric dependencies of the FOM with the ROM projection basis $\varvec{V}$. The model dependencies are expressed in Eq. (1) and captured in variable $\varvec{p}$, whereas the relation to $\varvec{V}$ from Eq. (3) is expressed through the coefficients $\varvec{X}$ from Eq. (7).

### VpROM: generating new bases

**Figure 3:**
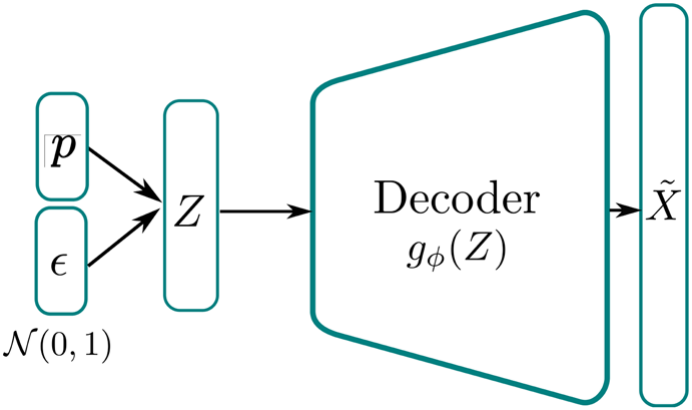
Architecture of the cVAE when used for basis generation: sample latent vectors are taken from the prior distribution $\epsilon$ and concatenated with the conditioning vector $\varvec{p}$ before these latent vectors are decoded to find the generated basis coefficients $\varvec{X}$ from Eq. (7).

In this work, we propose to train the cVAE for creating a generative model, which can be sampled in order to produce the reduced basis coefficients $\varvec{X}_i$ from Eq. (7) for a given parameter vector $\varvec{p}_i$. In which the parameters either reflect certain properties of the system or of the loading applied. Concretely, once a trained VAE is available, the encoder portion is no longer used and predictions are made purely using the decoder and the assumed variational distribution on the latent space. In this case, a diagonal Gaussian is used as shown in Fig. 3.

Hence, in order to sample from the decoder we simply take a draw, $\epsilon$, from the chosen prior distribution $p(\varvec{Z})$ and concatenate this draw with the given parameters $\varvec{p}$. We then pass this latent vector through the decoder, which results in a sample from the observation distribution $p_{\phi }(\varvec{X}\vert \varvec{Z},\varvec{p})$. This sample being a single draw from the distribution of the predicted coefficient values for a given parameter vector. Multiple such samples can then be taken by repeating this process in order to find quantities such as the mean and standard deviation of the predicted coefficients. This generative procedure emphasises the importance of minimising the KL divergence term in the loss function. If the KL loss is low then the the approximate posterior distribution $q_{\theta }(\varvec{Z}\vert X,p)$ better approaches the prior $p(\varvec{Z})$.

### Training the VpROM

As mentioned previously, we wish to train the cVAE to generate not the local bases themselves, but rather the coefficient matrices $\varvec{X}_i$ as introduced in Eq. (7), which are then used to generate the local ROM subspaces from a global basis. To do this, we require training pairs of parameter vectors $\varvec{p}_i$ and corresponding coefficient matrices $\varvec{X}_i$. These training pairs are created by first sampling the parameter vector with Latin Hypercube Sampling. Each of these training vectors is then used as model/input parameters for a FOM snapshot. The generated snapshots are then used to assemble the local bases $\varvec{V}_i$ and global basis $\varvec{V}_{global}$ respectively, and hence the coefficient matrices, using the procedure described in subsection "CpROM: Treatment of parametric dependencies via local Basis Coefficients Interpolation". After initial efforts, it was decided that a separate cVAE would be trained for each column of the coefficient matrix $\varvec{X}_i$. This was considered to be reasonable as each column of $\varvec{X}_i$ relates to a different retained POD mode, which ought to be mutually orthogonal. Further, the separate consideration of each column results in improved performance of the cVAE in generating new bases.

To prepare the data for training, the individual columns $\tilde{\varvec{X}}_{c}$, $c=1,...,r$ of the coefficient matrix $\varvec{X}\in {\textrm{R}}^{\tilde{r}*r}$ are taken as vectors and are paired with the parameter vectors $\varvec{p}_i$ for training. Further, the parameter values are normalised between $-1$ and 1 and the coefficient vectors are normalised, as shown in Eq. (15). The coefficient vectors $\tilde{\varvec{X}}_c$ are normalised using the application of a natural logarithm; this offers the advantage of rendering the amplitude of the vector components more similar and hence preventing extreme values from dominating the cost function. The addition of the constant 2 was required such that all the values were greater than zero in order to avoid an error in the logarithm operation.

15 $$\begin{aligned} \tilde{\varvec{X}}_c^{norm} = \ln {(\tilde{\varvec{X}}{_c}+2)} \end{aligned}$$

The models were all built and trained using Tensorflow and the Adam algorithm^74^. In training the cVAE, the architecture of the network, the number, and size of layers, and the activation functions used, must also be chosen, these can, however, be treated as hyperparameters and can be optimised according to common methods such as grid search. The network architecture has a massive effect on the expressive power and generalisation of the model. All trained models made use of only dense and dropout layers. Dense layers are a traditional fully connected feedforward neural network layer. Between each of the dense layers, a dropout layer was inserted. Dropout is a technique used for regularising deep neural networks, according to which - during each training update - a certain percentage (the dropout percentage) of activation values of a given layer are set to zero. This technique has been shown to improve generalisation performance of deep neural networks^75^. As such, the architectural hyperparameters to be optimised for each model include the number of dense layers in the encoder and decoder, the number of neurons in these layers, the activation function used by these layers, and the amount of dropout included between each layer. The size of the bottleneck layer, or in other words, the number of latent variables driving the process is also key for reduction. Noteworthy is, however, that in this case it was found that the application of dropout was not beneficial to the performance; as such, this was not used in any of the finally implemented models.

## Results

In this section, all aspects relevant to the performance of the proposed framework are validated on two case studies featuring parametric dependencies in system properties and excitation traits. The proposed cVAE-boosted ROM is firstly validated on a nonlinear benchmark simulator of a two-story shear frame featuring hysteretic joints^76^, and then on a larger scale simulation, featuring computational plasticity, which is based on the NREL reference 5-MW wind turbine tower^77^.

As already mentioned in section "Parametric reduced order modelling", we offer a comparison across alternate parametric ROM configurations in order to offer a comprehensive discussion on the potential and performance limits of the suggested framework. The first parametric ROM configuration refers to the MACpROM, as presented in subsection "MACpROM: treatment of parametric dependencies via clustering". This employs a MAC-guided clustering approach on the local POD bases. Next, the CpROM presented in subsection "CpROM: treatment of parametric dependencies via local basis coefficients interpolation" is evaluated, following the local basis coefficients interpolation approach in^55^. These two ROMs are assembled employing existing state-of-the-art approaches and serve comparison purposes. The last two parametric ROMs are derived based on the cVAE framework proposed here to inject parametric variability in the local projection bases. We evaluate the performance of the proposed cVAE-boosted ROM, termed *VpROM*, both with and without the inclusion of hyper-reduction, which is described in subsection "Hyper-reduction". The notation and configuration of these five schemes are summarised in Table 2 for reference. In what follows, we present different case studies comparing the performance of these different schemes.

**Table 2:** Reference table for compared ROMs.

Model reference name	Description
FOM	The full order finite element model
MACpROM	Pool of local bases and MAC-guided clustering. The approach is described in subsection "MACpROM: treatment of parametric dependencies via clustering".
CpROM	Formulation of coefficients $\varvec{X}$ for each local basis and interpolation, as presented in subsection "CpROM: treatment of parametric dependencies via local basis coefficients interpolation".
*VpROM*	The cVAE-boosted ROM as presented in section "VpROM: Coupling of Generative Models with projection-based ROMs"
HP-*	The respective * ROM additionally equipped with hyper-reduction

Regarding computational timings, the validation simulations of the presented examples are implemented using an in-house built FE code, based on the suggestions by^78^ and tested on a workstation equipped with an 11^th^ Gen Intel(R) Core(TM) i7-1165G7 processor, running at 2.80GHz, and 32GB of memory. In addition, the computational speed-ups reported do not include the training phase, or offline cost, of the ROMs and refer to the average acceleration obtained for every single model evaluation in prediction, or online mode. For the purpose of constructing Real Time Digital Twins, it is this quantity (the evaluation time) that holds the critical role of real-time evaluation. Thus, the reported computational time is averaged over all FOM or ROM evaluations of each respective set of configurations (training or testing).

The performance of the various frameworks in terms of reproducing the time history responses of the respective dynamic validation case studies is reported as follows:

16 $$\begin{aligned} err_q = \dfrac{\sqrt{\sum \limits _{i \in \tilde{N}_{\text {DOF}}} \sum \limits _{j \in \tilde{N}_{t}} \left( q_{i}^{j}-\tilde{q}_{i}^{j}\right) ^2}}{\sqrt{\sum \limits _{i \in \tilde{N}_{\text {DOF}}} \sum \limits _{j \in \tilde{N}_{t}} \left( q_{i}^{j}\right) ^2}} \times 100 \% \end{aligned}$$

where $\tilde{N}_{\text {DOF}}$ represents a set of DOFs selected for response comparison, $\tilde{N}_{t}$ a set of selected time steps, $q_{i}$ is the FOM quantity of interest at DOF *i*, and $\tilde{q}_{i}$ is the respective inferred value computed using the ROM approximation.

### Two story shear frame with hysteretic links

As an initial example, we consider a FE model of a three-dimensional two-story shear frame with nonlinear nodal couplings, each exhibiting a Bouc-Wen hysteretic nonlinearity^79^. This example is chosen as a demonstrative case study due to the inherent ability of the simulator to model multiple simultaneously activated instances of nonlinearity, thus challenging the precision of any derived ROM. Because this is a low dimensional example, we use it with the main purpose of assessing the accuracy of the respective ROMs from Table 2, as the model is rather trivial to demonstrate any substantial computational savings. The capacity of the various parametric ROMs in terms of accelerating model evaluations is documented in the next case study, featuring a large-scale wind turbine tower.

**Figure 4:**
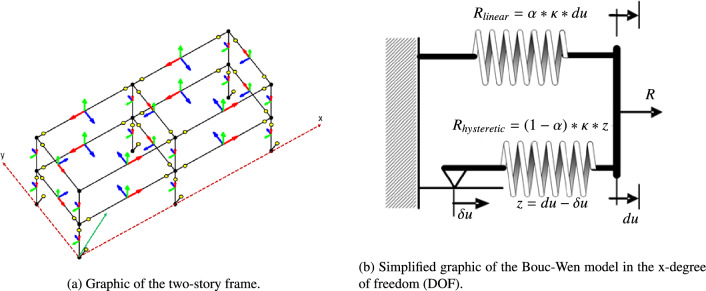
Graphic of the frame setup and illustration of the nonlinear mechanism in its links. The green arrow indicates the direction of ground motion, whereas the colored arrows the orientation of the beam elements.

A graphical illustration of the setup of the shear frame is visualized in Fig. 4a, where the hysteretic links assume no length, although the virtual nodes are depicted within a distance from the reference node in Fig. 4a for demonstration purposes. The respective model files that allow for results reproduction can be found in^76^, as this example has been published as a benchmark multi-degree of freedom nonlinear response simulator. Regarding material properties, the case study follows the template configuration^80^. Specifically, steel HEA cross-sections have been used for all beam elements, whereas the structure is assembled using two frames along axis *x*, each of $l = 7.5\,m$ length and one of $w = 6\,m$ along the width. In addition, each story has a height of $h = 3.2\,m$.

A Bouc-Wen formulation has been utilised to model the behavior of the nonlinear joints: this reflects a smooth hysteretic model, often adopted for modeling material nonlinearity^81^. Therefore, based on the benchmark description in^76^, a Bouc-Wen model is introduced at every DOF of every nodal coupling to simulate the total restoring force $\textbf{R}$ of each joint. An example illustration of the nonlinear mechanism in the longitudinal x-DOF is provided in Fig. 4b. The Bouc-Wen link models $\textbf{R}$ as a superposition of a linear and a nonlinear term, represented by the two springs in Fig. 4b. The linear and nonlinear terms, or springs, depend on the instantaneous nodal response $\delta u$ and the hysteretic, and thus history-dependent, component of the response *z*, respectively. In turn, the respective vectorized mathematical formulation for all DOFs of the link reads:

17 $$\begin{aligned} \textbf{R} = \textbf{R}_{linear} + \textbf{R}_{hysteretic} = \alpha *k*\textbf{du} + (1-\alpha )*k*\textbf{z} \end{aligned}$$

where $d\textbf{u}$ represents the nodal displacement, and $\alpha ,k$ are traits characterizing the Bouc-Wen model on each link. Regarding their physical interpretation, $\alpha$ represents the characteristic post-yield to elastic stiffness reaction for each link, whereas *k* is the corresponding stiffness coefficient. The variable $\textbf{z}$ stands for the hysteretic portion of the elongation, or displacement in general, and controls the hysteretic forcing. It obeys the following:

18 $$\begin{aligned} \dot{\textbf{z}} = \frac{A \textbf{d}\dot{\textbf{u}} - \nu (t) (\beta |\textbf{d}\dot{\textbf{u}}|\textbf{z}|\textbf{z}|^{w-1}-\gamma \textbf{d}\dot{\textbf{u}}|\textbf{z}|^{w})}{\eta (t)} \end{aligned}$$

19 $$\begin{aligned} \nu (t) = 1.0 + \delta _{\nu } \epsilon (t), \quad \eta (t) = 1.0 + \delta _{\eta } \epsilon (t), \quad \epsilon (t) = \int _0^{t} \textbf{z}\textbf{d}\dot{\textbf{u}} dt \end{aligned}$$

where the shape, smoothness, and overall amplitude of the hysteretic curve that characterises the dynamic behavior of each joint is determined by the Bouc-Wen parameters $\beta , \gamma , w$, and *A* respectively. The terms $\nu (t), \eta (t)$ are introduced to capture strength deterioration and stiffness degradation effects via the corresponding coefficients $\delta _{\nu }$ and $\delta_{\eta}$. In turn, their evolution in time depends on the absorbed hysteretic energy, $\epsilon (t)$. This representation allows for a structural dynamics simulator, which is parametrised with respect to system properties and traits of the joints’ behavior. For a more detailed elaboration on the physical connotations of the Bouc-Wen model parameters in terms of yielding, softening, and hysteretic behavior effects, the reader is referred to^76,82^.

This parameterised shear frame simulator is selected due to its ability to model a variety of nonlinear dynamic effects that dominate the response and are dependent on the parametric configuration of the model. In the presented case studies, the parameters defining the structure itself and those defining the acting loads can significantly affect the response. The parameter set includes the forcing signal’s temporal and spectral characteristics, the frame’s material properties, and the traits that dictate the hysteretic effects on the joints. The six parameters employed in this example are summarised in Table 3.

**Table 3:** Two-story frame: Range of the parameter values of the ROM. All parameters follow a uniform distribution. .

Parameter	$\alpha$	*k*	*Amp*	$f_{but}$	*E*	$\delta _{\eta }$
Range	[0.25,0.50]	[0.8,1.2]$\times 10^8$	[1.5,3.0]$\times 10^6$	[5,15] Hz	[185,235] GPa	[0.25, 0.75]

First, uncertainty is introduced in the material properties of the system by treating the Young modulus of elasticity *E* as a parameter of the model. Its range is summarized in Table 3. In addition, three traits of the nonlinear joints of the shear frame are parametrically to model and simulate various qualities and shapes of the corresponding hysteretic behavioral curves. Specifically, parameters $\alpha$ and *k* in Eq. (17) and parameter $\delta _n$ in Eq. (18) are injected as dependencies in the derived ROM. The numerical range for each parameter is also provided in Table 3.

Forcing is applied to the frame system as a base excitation scenario representing an earthquake. The force is applied at an angle of $\theta = \pi /4$ with respect to the x-axis, as depicted in Fig. 4a. To produce a parameterised version of the excitation, a white noise template accelerogram is used as a reference. This template signal is then passed through a second-order butterworth low-pass filter and multiplied with an amplitude factor to produce the actual accelerogram of the motion imposed on the system. The amplitude coefficient *Amp* and the frequency of the filter $f_{but}$ are treated as dependencies. Thus, due to the dependencies of the model both in system parameters and excitation traits, the dynamic system under consideration exhibits substantially different behavior depending on the chosen parametric configuration.

The numerical study has been designed in such a way to validate the requirement to inject dependencies in the derived surrogate while making use of the ability of the simulator to output a variety of nonlinear behaviors, thus challenging the accuracy limit of the ROMs.

All ROMs as referenced in Table 2 are implemented here, employing the same training scheme of fifty samples drawn using LHS. The corresponding performance measures for each ROM are evaluated on a validation set of five hundred samples, drawn using an LHS with a different seed. Regarding the low-order dimension, $r=16$ modes are retained for each local basis $\varvec{V}$ and $\tilde{r}=200$ modes for $\varvec{V}_{global}$ in Eq. 7.

**Figure 5:**
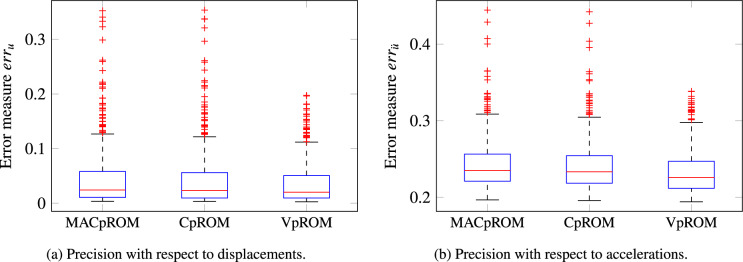
Box plots reporting the accuracy of capturing the displacement and acceleration time histories. The distributions of the respective error measures $err_u,err_{\ddot{u}}$ from Eq. (16) are visualised along with the respective median (red line) and outliers (red crosses).

A detailed evaluation of the accuracy for the implemented ROMs of Table 2 is presented in Fig. 5. The precision of the respective surrogates is evaluated with respect to two measures, namely $err_u$ and $err_{\ddot{u}}$ of Eq. (16) that correspond to the error on capturing the displacement and acceleration time histories respectively. The boxplots provide a visualisation of the ability of each ROM to capture the FOM response both in terms of displacements and accelerations, whereas the respective values are also reported in Table 4. Although the overall precision is relatively low, this example merely serves to offer a comparison that deliberately employs a relatively wide domain of parameters, in order to excite substantially different dynamic behavior.

**Table 4:** Performance for the ROMs from Table 2.Accuracy is reported with respect to displacements ($err_u$) and accelerations($err_{\ddot{u}}$). Hyper-reduction is not implemented, and timings exclude the training phase and refer to online model evaluations.

	Median		Maximum		Efficiency	
	Error		Error		Speed-Up	CPU
	$err_u$	$err_{\ddot{u}}$	$err_u$	$err_{\ddot{u}}$	Factor	Timing
FOM	–	–	–	–	1.00	53 (s)
MACpROM	2.42$\%$	35.23$\%$	23.52$\%$	44.44$\%$	2.22	24 (s)
CpROM	2.35$\%$	35.34$\%$	23.33$\%$	44.22$\%$	2.10	25 (s)
VpROM	2.20$\%$	19.62$\%$	22.57$\%$	33.81$\%$	2.41	22 (s)

Nevertheless, in Fig. 5 and Table 4 the MACpROM implemented exhibits similar accuracy with the reference CpROM, in terms of approximating both the displacement and the acceleration time histories. The respective median error and the boxplot quartiles almost coincide, whilst both approaches seem to deliver a similar distribution of outliers. The proposed *VpROM* on the other hand achieves a substantially improved performance. The outliers are fewer, the respective discrepancy for the outlier samples is substantially lower, and the visualised distribution has a lower median and maximum error.

**Figure 6:**
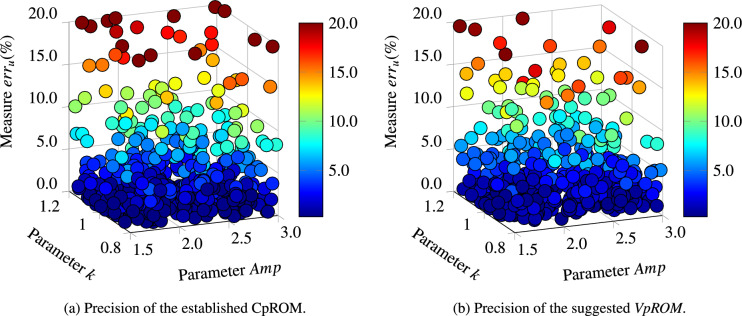
Parameter Visualisation of the error distribution of Eq. (16) for displacement time histories ($err_u$). The proposed *VpROM* is compared with CpROM, as the MACpROM delivers a slightly worse performance as indicated in Fig. 5. All samples with errors greater than $20\%$ are depicted in the $20\%$ color level for a clearer comparison.

A further comparative visualisation of the accuracy for the implemented ROMs is provided in Fig. 6. An example projection plane has been chosen for demonstration purposes and the validation measure is depicted on the vertical axis and via the color scale. Similar to Fig. 5a, the $err_u$ is visualised in Fig. 6 as a representative measure of the ROMs ability to reproduce the displacement time histories of the FOM. In addition, all samples with errors greater than $20\%$ are depicted in the $20\%$ color level for better scaling and a clearer comparison. Since the established CpROM and the MACpROM deliver similar precision with respect to displacements in Fig. 5a, the *VpROM* suggested in this study is compared only with CpROM in Fig. 6 for the sake of a clearer demonstration.

As already highlighted, the *VpROM* captures the dynamics across the domain of parametric inputs with an overall superior precision and fewer accuracy outliers than CpROM or MACpROM. This is visualised in Fig. 6 through the fewer circles located in the dark red region for the *VpROM* and the substantially fewer evaluations colored outside the blue-to-green spectrum. Thus, despite a few outliers, the overall accuracy of the framework remains superior to the compared established alternatives for physics-based MOR.

**Figure 7:**
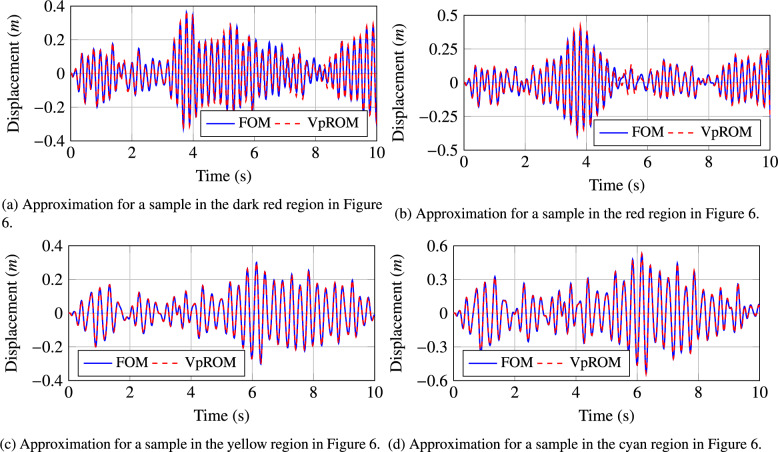
Visualisation of the different levels of the approximation quality achieved using the *VpROM*. The *VpROM* estimation is reported for various response patterns the system exhibits depending on its parametric features.

In Fig. 7, a more detailed evaluation of the approximation quality achieved by the *VpROM* is illustrated. Specifically, the time history estimation is depicted for different levels of precision to visualise and validate the overall performance of the *VpROM*. One sample from each family of sample points as captured by the color scale in Fig. 6 is presented. The *VpROM* approximation is visualised for various response patterns to highlight the ability of the surrogate model to infer dynamic behaviors dominated by different effects as modeled via the variety of shapes of the hysteretic curves on the links. The *VpROM* is shown to deliver a robust approximation in a complex example with a rich dynamic behavior represented by several different shapes and amplitudes of the hysteretic curves characterizing the behavior of the nonlinear joints.

**Figure 8:**
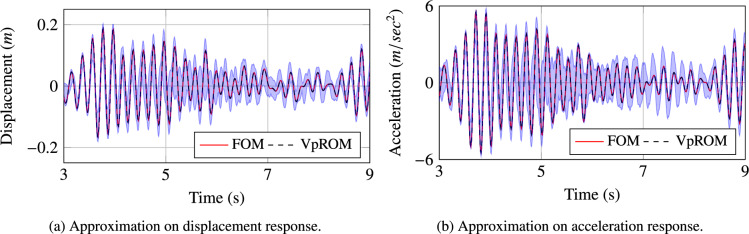
Average quality of the *VpROM* approximation. Evaluation is performed on the degree of freedom with the maximum absolute response. The shaded area quantifies the uncertainty of the response inference.

Beyond offering a robust reduction framework that is shown to generalise across parametric configurations, the derived *VpROM* offers the potential of quantifying the uncertainty in the respective estimations. This is due to the latent space of the cVAE component being trained to approximate a given variational distribution; thus, by sampling this variational distribution, the uncertainty on the predicted local basis coefficients may be captured in addition to simply mean estimates. Naturally, by using only the mean predicted values of the coefficients, significant information is lost regarding the uncertainty of these values. Hence, it is worth considering utilising this technique for uncertainty estimation on the response of the ROM for each sample. The uncertainty captured by the cVAE is that associated with the coefficients of the reduction basis generated by the cVAE. To estimate the uncertainty on the model output, multiple samples are drawn from the cVAE for a given parameter vector, parallel evaluations of the *VpROM* are then performed with each of the sampled bases. The uncertainty on the coefficients is then propagated to the *VpROM* prediction by examining the distribution of the responses from these parallel simulations. These distributions can then be quantified and confidence bounds extracted, this can provide increased utility for many problems in structural dynamics.

A visualisation of the respective output is provided in Fig. 8. This figure plots the performance of the *VpROM* in both displacement and acceleration for the parameter vector, which had the median error value within the testing set. The respective shaded area represents the confidence bounds of the inference scheme, evaluated by sampling the predicted distributions of the latent space 40 times and propagating the response using the respective local bases assembled by the decoder for each of these 40 sampled vectors. The shaded area encompasses the maximum and minimum values at each time point for the 40 simulations carried out. The respective confidence bounds are relatively tight around the model’s response, indicating a robust and reliable surrogate.

**Figure 9:**
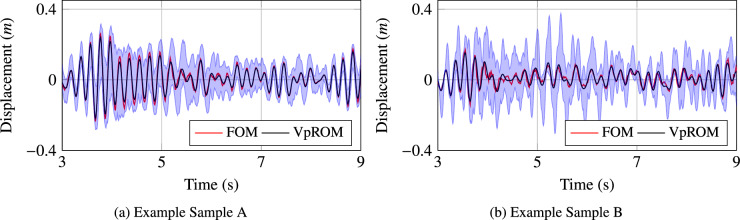
Quality of the *VpROM* displacement response approximation and range of the confidence bounds for parametric samples outside the training domain. Both samples lie near the limits of the domain defined in Table 3. Example sample A is $[\alpha ,k,Amp,f_{but},E,\delta _{\eta }]$=[0.47, 0.89, 1.52, 14.93, 185, 0.63], whereas example sample B is [0.48, 0.81, 2.95, 5.33, 228, 0.60].

To further demonstrate and challenge the applicability limits of the derived *VpROM*, its performance is validated in two example samples located beyond the training region. Both examined samples lie near the limits of the domain defined in Table 3, thus representing potential operational configurations of the system. The quality of the *VpROM* approximation, along with the respective uncertainty bounds, is illustrated in Fig. 9. As expected, the shaded region indicating the confidence of the prediction is considerably larger compared to Fig. 8, since the framework is extrapolating to testing samples located outside the training domain. In addition, the performance deterioration and the considerably increased uncertainty for example sample B reported in Fig. 9b, imply that sample B triggers a response containing complex and higher-order dynamics that are not well-represented in the training set. On the contrary, the latent distribution of the *VpROM* seems able to accurately infer the response for example sample A, including relatively tight confidence bounds, although sample A lies outside the training regime. This potentially indicates that the obtained uncertainty range does not necessarily increase only due to extrapolation but also depending on the complexity and higher-order dynamic effects represented in the extrapolation region.

Nevertheless, the respective average quality of the *VpROM* approximation as depicted in Fig. 8 indicates a high-precision physics-based surrogate, with the inherent ability to provide a quantification on the uncertainty of the respective estimations. To further evaluate the suitability of the proposed framework and demonstrate its utility in reducing computational toll, a large-scale example is discussed next.

### Wind turbine tower with plasticity

This section evaluates the performance of the suggested *VpROM* on a large-scale example based on the simulated dynamic response of the NREL 5-MW reference wind turbine tower^77^. Regarding the configuration of this case study, the interested reader is referred to^55^.

**Figure 10:**
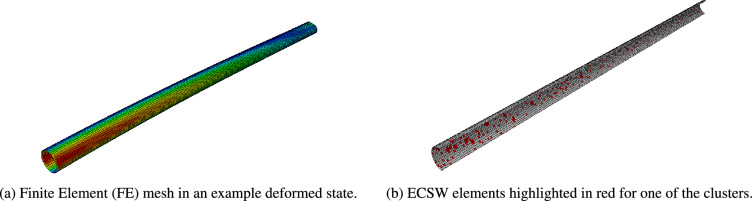
Wind turbine tower: FE model and example of ECSW mesh. For the ECSW elements a horizontal cut is depicted for visualization purposes.

In brief, the three-dimensional FE model of the monopile is visualized in Fig. 10a and features a circular cross-section, which is linearly tapered from the base to the top. The respective diameter and wall thickness are equal to 6*m* and 0.027*m* on the base and 3.87*m* and 0.019*m* on the top of the monopile. However, for simplification purposes, a constant thickness assumption is made throughout the tower, and 8170 shell elements are used. The wind turbine is assembled at the top of the monopile assuming a lumped mass scheme and regular beam elements, assembled through multi-point constraints to the tower. Regarding the material properties, steel is assumed, with $E_{steel}=210 GPa$ and a density of $\rho =7850 kg/m^3$ and a nonlinear constitutive law, which is characterised by isotropic von Mises plasticity.

Although the employed stress-strain relation might seem relatively simple, this problem features an extensive yielding domain ($\approx 30\%$ of the height), additional model uncertainties, and a stochastic excitation that increase the complexity and pose certain requirements when deriving a high precision ROM. In addition, this large-scale case study is used to demonstrate the efficiency of hyper-reduced ROMs, which allow for a substantial reduction in the overall computational toll. For this reason, the hyper-reduced variant of all ROMs, termed as *HR-VpROM* for example in Table 2, is validated herein.

**Table 5:** Range of the parametric values of the implemented ROMs. The $E_{ref}$ refers to the typical modulus assigned to each material of the model.

Parameter	Excitation amplitude *A*	Yield stress $\sigma _{VM}$	Young modulus *E*
Range	[2.50, 3.75]	[375, 450] (MPa)	[0.80, 1.20]$\times E_{ref}$ (GPa)

Regarding the parametric dependencies, the Kobe earthquake accelerogram is utilised as a ground motion scenario, parameterised with respect to its amplitude *A*. The yield stress $\sigma _{VM}$ and the Young modulus of elasticity *E* are also varied. The range of these parametric dependencies is summarised in Table 5. The training and validation domain is designed using LHS sampling, similar to subsection "Two story shear frame with hysteretic links". In this case study, a low-order dimension of $r=4$ is chosen, while $\tilde{r}=32$ global modes are retained for $\varvec{V}_{global}$ in Eq. (7), whereas the $\tau$ parameter for the ECSW hyper-reduction technique discussed in subsection "Hyper-reduction" is set to $\tau =0.01$. The ability of the proposed *HP-VpROM* to accurately infer response fields that are relevant for dynamic structural systems, while providing accelerated model evaluations is exhibited herein.

**Figure 11:**
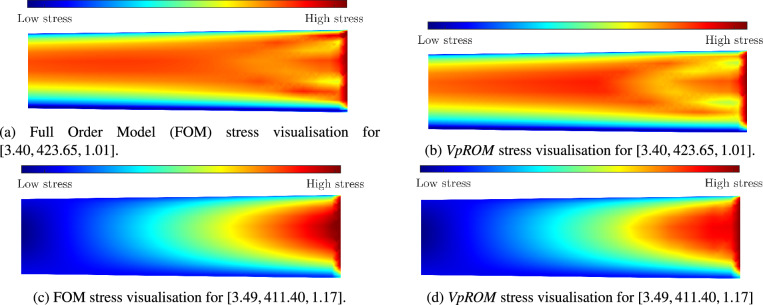
Visualisation of the approximation of internal stresses achieved by the proposed *VpROM* using nodal averaging. Only the yielding domain is visualised, which extends to one-third of the total height.

In Fig. 11 a visualisation of the *HP-VpROM* approximation for the internal stress field in the yielding domain is provided for two validation examples, which feature different dynamic behavior. The respective high-fidelity field is also visualised via the FOM for reference purposes. Stresses and strains are important metrics to be monitored in many structural applications; thus, the ability to capture their distribution accurately is often of critical importance. Despite the minor discrepancies observed, the overall quality of the *HP-VpROM* approximation illustrated in Fig. 11 indicates an effective low-order representation, able to deliver high precision estimates of stress state distributions. This exemplifies the potential utility of the proposed *HP-VpROM* in condition monitoring, fatigue, or damage localisation.

**Table 6:** Performance of the hyper-reduced ROMs from Table 2. Two validation samples are presented, along with the median and max error measures from Eq. (16). Efficiency is also reported, excluding the offline cost.

	Sample		Sample		Average		Maximum		Efficiency	
	[2.5,375,0.80]		[3.75,450,1.2]		Error		Error		Speed-Up	CPU
	$err_u$	$err_{\ddot{u}}$	$err_u$	$err_{\ddot{u}}$	$err_u$	$err_{\ddot{u}}$	$err_u$	$err_{\ddot{u}}$	Factor	Timing
FOM	–	–	–	–	–	–	–	–	1.00	3990 (s)
HP-MACpROM	2.01%	6.44%	1.54%	5.88%	2.34$\%$	6.24$\%$	8.89$\%$	7.64$\%$	35.40	112 (s)
HP-CpROM	1.44%	6.48%	1.06%	5.87%	2.05$\%$	6.22$\%$	5.09$\%$	7.48$\%$	36.69	109 (s)
HP-VpROM	0.51%	6.44%	0.84%	5.89%	0.91$\%$	5.98$\%$	1.88$\%$	7.13$\%$	35.67	112 (s)

A more comprehensive summary of all aspects of the ROMs’ performance is provided in Table 6. Specifically, the average and maximum error measures of the respective approximations on displacement and acceleration time histories are summarised for all hyper-reduced surrogates of Table 2. In addition, the precision is reported for two example validation samples in the extreme regions of the input domain for reference.

**Figure 12:**
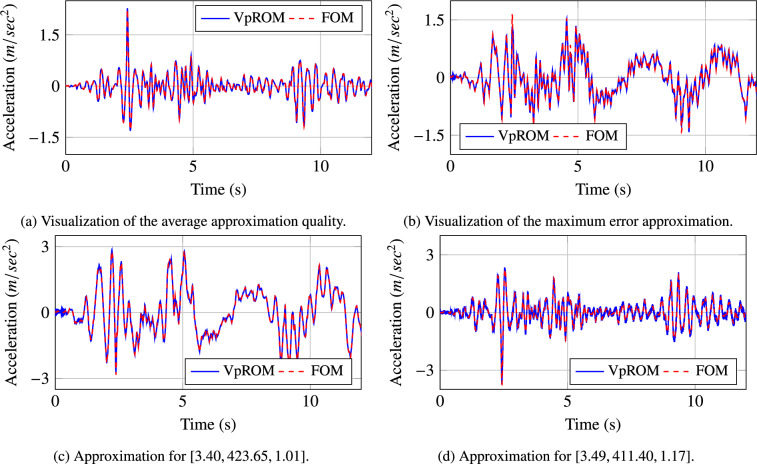
Visualisation of the different levels of the approximation quality achieved using the *HP-VpROM*. The estimation is reported for various response patterns the system exhibits depending on its parametric features.

Similar to what was already observed in the previous case study in subsection "Two story shear frame with hysteretic links", the hyper-reduced variant of the *VpROM*, namely *HP-VpROM*, delivers a superior surrogate in terms of accuracy. The respective low maximum $err_u$ measure on capturing the displacements indicates a robust approximation, whereas the other two ROMs experience performance outliers where accuracy deteriorates significantly. At the same time, *HP-VpROM* achieves an average discrepancy lower than $1\%$, implying a high precision representation. Similar conclusions can be drawn by observing the respective measures on the two validation samples offered as additional examples. Regarding the inference of the acceleration response, the *VpROM* maintains its superior accuracy, although it seems only marginally better than the two alternative ROMs implemented.

The utility of the proposed *HP-VpROM* for applications, in which (near) real-time model evaluations are required is also documented in Table 6. The hyper-reduction technique, along with the ability of the ROM to propagate the dynamics in a proper low-order subspace achieves a substantial computational toll reduction and accelerated computations. The respective average speed-up factor $t_{FOM}/t_{ROM}$ reported in Table 6 implies significant savings in computational resources during model evaluations.

The reported performance measures highlight the fact that the proposed ROM framework remains robust and precise, also when coupled with hyper-reduction. The generative model injected guarantees the ability of the *HP-VpROM* to capture different dynamic trends in the response and avoid accuracy outliers. This is indicatively visualised in Fig. 12, where the acceleration response of the full-order model and the respective *HP-VpROM* approximation is illustrated for validation samples located near the edges of the input domain.

**Figure 13:**
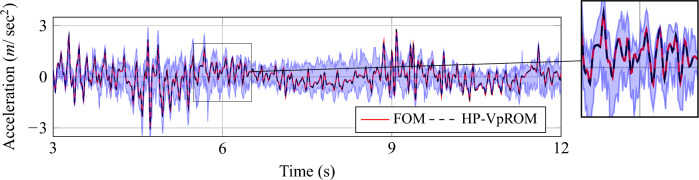
Quality and confidence bounds of the *HR-VpROM* approximation. Evaluation is performed on the degree of freedom with the maximum absolute response. The shaded area quantifies the uncertainty of the response inference.

The different patterns in the system’s behavior are demonstrated clearly, along with the ability of the assembled *HP-VpROM* to capture the different trends sufficiently accurately. Despite the minor discrepancies observed, especially when high-frequency components are present in the response, the HP-VpRO*M* maintains a robust performance with a high-quality approximation across the input domain.

The coupling of the ROM with a cVAE-based generative model offers an additional feature, namely the quantification of the uncertainty in the estimations. Relying on the probabilistic nature of the latent space of the assembled cVAE the proposed *HP-VpROM* comes with confidence bounds on its predictions. This is exemplified in Fig. 13, where the approximation for the acceleration is visualised for a representative validation sample. The shaded region in Fig. 13 represents the uncertainty of the respective prediction and may be used as a confidence measure during when using the ROM predictions this improves the utility of the scheme compared to determinstic methods.

## Limitations and concluding remarks

This work demonstrates the use of a cVAE-based ROM, termed as *VpROM*, as an extension to state of the art methods for generating local reduction bases for nonlinear parametric ROMs.

The following conclusions are drawn:

- cVAE neural networks can successfully be used to generate local bases for nonlinear parametric ROMs with high dimensional parameterisation and strongly nonlinear behaviour.
- The verification of the proposed scheme on a large-scale system results in significantly accelerated model evaluations, almost 40 times faster than the FOM. This speed-up excludes the training cost and refers to the average timing of online model evaluations.
- The cVAE can outperform current state of the art methods such as interpolation and clustering algorithms in terms of precision of the ROM.
- The *VpROM* formulation offers the additional benefit of encoding the uncertainty in the predicted local bases and the ability to propagate this in the predicted response.

The newly developed method is demonstrated on two simulated nonlinear systems, which are parameterised in terms of both system and loading traits. The first example demonstrates the viability of the method for a system of high dimensional parametric dependency exhibiting strongly varying nonlinear behaviour. The second example verifies the proposed scheme on a large-scale system, with the inclusion of hyper-reduction, in order to demonstrate the utility of the method for hyper-accelerated model evaluations. In both examples, the potential of the method for quantifying uncertainty on its estimates is also demonstrated.

the main limitation of the method in comparison to current methodologies is the relative complexity of the training process of the cVAE models. Owing to their very flexible nature neural network methods, such as VAEs, comprise a relatively high number of hyper-parameters that must be tuned for optimal performance. When training the *VpROM*, it is necessary to select such hyper-parameters, which include for instance, the number of layers in the network and the number of neurons in each of these layers, the activation functions used in the network, and the learning rate of the optimisation algorithm. There exist a number of heuristics for choosing such parameters, such as grid search, yet it remains worth highlighting that this process requires more effort than is required for other state-of-the-art methods exploiting clustering or interpolation methods.

## Supplementary Information

Supplementary Information.

## Data Availability

The data that support the findings of this study are available from the authors, TS, KV, upon reasonable request.

## References

[1] Ebrahimian, H., Astroza, R., Conte, J. P. & de Callafon, R. A. Nonlinear finite element model updating for damage identification of civil structures using batch Bayesian estimation. *Mech. Syst. Signal Process.* **84**, 194–222 (2017).

[2] Rosafalco, L., Torzoni, M., Manzoni, A., Mariani, S. & Corigliano, A. Online structural health monitoring by model order reduction and deep learning algorithms. *Comput. Struct.* **255**, 106604 (2021).

[3] Edington, L., Dervilis, N., Abdessalem, A. B. & Wagg, D. A time-evolving digital twin tool for engineering dynamics applications. *Mech. Syst. Signal Process.* **188**, 109971 (2023).

[4] Solman, H., Kirkegaard, J. K., Smits, M., Van Vliet, B. & Bush, S. Digital twinning as an act of governance in the wind energy sector. *Environ. Sci. Policy* **127**, 272–279 (2022).

[5] Lüthen, N., Marelli, S. & Sudret, B. A spectral surrogate model for stochastic simulators computed from trajectory samples. *Comput. Methods Appl. Mech. Eng.* **406**, 115875 (2023).

[6] Sudret, B., Marelii, S., & Wiart, J. Surrogate models for uncertainty quantification: An overview. In * 2017 11th European Conference on Antennas and Propagation (EUCAP)*, Paris, France, 793–797 (2017).

[7] Guo, M. & Hesthaven, J. S. Data-driven reduced order modeling for time-dependent problems. *Comput. Methods Appl. Mech. Eng.* **345**, 75–99 (2019).

[8] Vlachas, P. R., Byeon, W., Wan, Z. Y., Sapsis, T. P. & Koumoutsakos, P. Data-driven forecasting of high-dimensional chaotic systems with long short-term memory networks. In * Proceedings of the Royal Society A*, 474, (May 2018). 10.1098/rspa.2017.0844 PMC5990702 29887750

[9] Brink, A. R., Najera-Flores, D. A. & Martinez, C. The neural network collocation method for solving partial differential equations. *Neural Comput. Appl.* **33**, 5591–5608 (2021).

[10] Zhang, R., Liu, Y. & Sun, H. Physics-informed multi-LSTM networks for metamodeling of nonlinear structures. *Comput. Methods Appl. Mech. Eng.* **369**, 2323–2326 (2020).

[11] Garland, A., Potter, K. & Smith, M. Feature anomaly detection system (FADS) for intelligent manufacturing. arXiv:2204.10318 (2022).

[12] Peherstorfer, B. & Willcox, K. Data-driven operator inference for nonintrusive projection-based model reduction. *Comput. Methods Appl. Mech. Eng.* **306**, 196–215 (2016).

[13] Carlberg, K. & Farhat, C. A compact proper orthogonal decomposition basis for optimization-oriented reduced-order models. In *12th AIAA/ISSMO Multidisciplinary Analysis and Optimization Conference*, Victoria, British Colombia, Canada, AIAA. (2008).

[14] Najera-Flores, D. A. & Todd, M. D. A structure-preserving neural differential operator with embedded Hamiltonian constraints for modeling structural dynamics. *Comput. Mech.*10.1007/s00466-023-02288-w (2023).

[15] Qian, E., Kramer, B., Peherstorfer, B. & Willcox, K. Lift & learn: Physics-informed machine learning for large-scale nonlinear dynamical systems. *Phys. D: Nonlinear Phenom.* **406**, 132401 (2020).

[16] Benner, P., Ohlberger, M., Cohen, A. & Willcox, K. Model reduction and approximation: theory and algorithms. *SIAM* (2017).

[17] Gobat, G., Opreni, A., Fresca, S., Manzoni, A. & Frangi, A. Reduced order modeling of nonlinear microstructures through proper orthogonal decomposition. *Mech. Syst. Signal Process.* **171**, 108864 (2022).

[18] Chinesta, F., Keunings, R. & Leygue, A. *The Proper Generalized Decomposition for Advanced Numerical Simulations: A Primer* (Springer, 2013).

[19] Chinesta, F., Ammar, A., Leygue, A. & Keunings, R. An overview of the proper generalized decomposition with applications in computational rheology. *J. Non-Newton. Fluid Mech.*, **166**(11), 578–592 (2011). XVIth International Workshop on Numerical Methods for Non-Newtonian Flows.

[20] Niroomandi, S. *et al.* Real-time simulation of biological soft tissues: A PGD approach. *Int. J. Numer. Methods Biomed. Eng.* **29**(5), 586–600 (2013). 10.1002/cnm.2544 23495247

[21] Agathos, K., Bordas, S. P. A. & Chatzi, E. Parametrized reduced order modeling for cracked solids. *Int. J. Numer. Meth. Eng.* **121**(20), 4537–4565 (2020).

[22] Christensen, E. A., Brøns, M. & Nørkær Sørensen, J. Evaluation of proper orthogonal decomposition-based decomposition techniques applied to parameter-dependent nonturbulent flows. *SIAM J. Sci. Comput.* **21**(4), 1419–1434 (1999).

[23] Georgaka, S., Stabile, G., Rozza, G. & Bluck, M. J. Parametric POD-Galerkin model order reduction for unsteady-state heat transfer problems. *Commun. Comput. Phys.* **27**(1), 1–32 (2019).

[24] Kerschen, G., Golinval, J. C., Vakakis, A. F. & Bergman, L. A. The method of proper orthogonal decomposition for dynamical characterization and order reduction of mechanical systems: An overview. *Nonlinear Dyn.* **41**, 147–169 (2005).

[25] Zimmermann, R., & Debrabant, K. Parametric model reduction via interpolating orthonormal bases. In * European Conference on Numerical Mathematics and Advanced Applications*, 683–691. (Springer, 2017).

[26] Amsallem, D., Zahr, M. J. & Farhat, C. Nonlinear model order reduction based on local reduced-order bases. *Int. J. Numer. Meth. Eng.* **92**(10), 891–916 (2012).

[27] Mahdiabadi, M. K., Tiso, P., Brandt, A. & Rixen, D. J. A non-intrusive model-order reduction of geometrically nonlinear structural dynamics using modal derivatives. *Mech. Syst. Signal Process.* **147**, 107126 (2021).

[28] Long, W., Tiso, P., Tatsis, K., Chatzi, E. & van Keulen, F. A modal derivatives enhanced Rubin substructuring method for geometrically nonlinear multibody systems. *Multibody Sys. Dyn.* **45**(1), 57–85 (2019). 10.1007/s11044-018-09644-2 PMC6394421 30881201

[29] Kapteyn, M. G., Knezevic, D. J., Huynh, D. B. P., Tran, M. & Willcox, K. E. Data-driven physics-based digital twins via a library of component-based reduced-order models. *Int. J. Numer. Meth. Eng.* **123**(13), 2986–3003 (2022).

[30] Quarteroni, A. *et al.**Reduced Order Methods for Modeling and Computational Reduction* Vol. 9 (Springer, 2014).

[31] Amsallem, D. & Haasdonk, B. PEBL-ROM: Projection-error based local reduced-order models. *Adv. Model. Simul. Eng. Sci.* **3**(1), 1–25 (2016).

[32] Morsy, A. A. Kast, M. & Tiso, P. A reduced order model for joint assemblies by hyper-reduction and model-driven sampling. arXiv:2204.12160 (2022).

[33] Haasdonk, B., Dihlmann, M. & Ohlberger, M. A training set and multiple bases generation approach for parameterized model reduction based on adaptive grids in parameter space. *Math. Comput. Model. Dyn. Syst.* **17**(4), 423–442 (2011).

[34] Rozza, G., Huynh, D. B. P. & Manzoni, A. Reduced basis approximation and a posteriori error estimation for stokes flows in parametrized geometries: Roles of the inf-sup stability constants. *Numer. Math.* **125**(1), 115–152 (2013).

[35] Paul-Dubois-Taine, A. & Amsallem, D. An adaptive and efficient greedy procedure for the optimal training of parametric reduced-order models. *Int. J. Numer. Meth. Eng.* **102**(5), 1262–1292 (2015).

[36] Grimberg, S., Farhat, C., Tezaur, R. & Bou-Mosleh, C. Mesh sampling and weighting for the hyperreduction of nonlinear Petrov-Galerkin reduced-order models with local reduced-order bases. *Int. J. Numer. Meth. Eng.* **122**(7), 1846–1874 (2021).

[37] Ghavamian, F., Tiso, P. & Simone, A. POD-DEIM model order reduction for strain-softening viscoplasticity. *Comput. Methods Appl. Mech. Eng.* **317**, 458–479 (2017).

[38] Cicci, L., Fresca, S. & Manzoni, A. Deep-hyromnet: A deep learning-based operator approximation for hyper-reduction of nonlinear parametrized PDEs. arXiv:2202.02658 (2022).

[39] Fresca, S. & Manzoni, A. POD-DL-ROM: enhancing deep learning-based reduced order models for nonlinear parametrized PDEs by proper orthogonal decomposition. *Comput. Methods Appl. Mech. Eng.* **388**, 114181 (2022).

[40] Goodfellow, I., Pouget-Abadie, J., Mirza, M., Xu, B., Warde-Farley, D., Ozair, S., Courville, A. & Bengio, Y. Generative adversarial nets. In: *Advances in Neural Information Processing Systems*. (Curran Associates, Inc., 2014).

[41] Kingma, D. P. & Welling, M. Auto-encoding variational bayes. In * 2nd International Conference on Learning Representations, ICLR 2014 - Conference Track Proceedings*, Banff, Alberta, Canada, ICLR. (2014).

[42] Bowman, S. R., Vilnis, L., Vinyals, O., Dai, A. M., Józefowicz, R. & Bengio, S. Generating sentences from a continuous space. * CoRR*. abs/1511.06349 (2015).

[43] Pagnoni, A., Liu, K. & Li, S. Conditional variational autoencoder for neural machine translation. arXiv:1812.04405 (2018).

[44] Kadurin, A. *et al.* The cornucopia of meaningful leads: Applying deep adversarial autoencoders for new molecule development in oncology. *Oncotarget* **8**(7), 10883–10890 (2018). 10.18632/oncotarget.14073 PMC5355231 28029644

[45] Sanchez-Lengeling, B. & Aspuru-Guzik, A. Inverse molecular design using machine learning: Generative models for matter engineering. *Science* **361**(6400), 360–365 (2018). 30049875 10.1126/science.aat2663

[46] Prakash, M., Krull, A., & Jug, F. Fully unsupervised diversity denoising with convolutional variational autoencoders. arXiv:2006.06072 (2020).

[47] Rosenbaum, D., Garnelo, M., Zielinski, M., Beattie, C., Clancy, E., Huber, A., Kohli, P., Andrew W. S., John, J. & Doersch, C., et al. Inferring a continuous distribution of atom coordinates from cryo-EM images using vaes. arXiv:2106.14108 (2021).

[48] Dhariwal, P. & Nichol, A. Diffusion models beat gans on image synthesis. In (eds M. Ranzato, A. Beygelzimer, Y. Dauphin, P.S. Liang, J. Wortman Vaughan) * Advances in Neural Information Processing Systems*, volume 34, 8780–8794. (Curran 572 Associates, Inc., 2021).

[49] Ho, J., Salimans, T., Gritsenko, A., Chan, W., Norouzi, M. & Fleet, D. J. Video diffusion models. arXiv:2204.03458 (2022).

[50] Tsialiamanis, G., Champneys, M. D., Dervilis, N., Wagg, D. J. & Worden, K. On the application of generative adversarial networks for nonlinear modal analysis. *Mech. Syst. Signal Process.* **166**, 108473 (2022).

[51] Mylonas, C., Abdallah, I. & Chatzi, E. Conditional variational autoencoders for probabilistic wind turbine blade fatigue estimation using supervisory, control, and data acquisition data. *Wind Energy* **24**, 1122–1139 (2021).

[52] Tatsis, K. E., Agathos, K., Chatzi, E. N. & Dertimanis, V. K. A hierarchical output-only Bayesian approach for online vibration-based crack detection using parametric reduced-order models. *Mech. Syst. Signal Process.* **167**, 108558 (2022).

[53] Agathos, K., Tatsis, K. E., Vlachas, K. & Chatzi, E. Parametric reduced order models for output-only vibration-based crack detection in shell structures. *Mech. Syst. Signal Process.* **162**, 108051 (2022).

[54] Boncoraglio, G. & Farhat, C. Active manifold and model-order reduction to accelerate multidisciplinary analysis and optimization. *AIAA J.* **59**(11), 4739–4753 (2021).

[55] Vlachas, K., Tatsis, K., Agathos, K., Brink, A. R. & Chatzi, E. A local basis approximation approach for nonlinear parametric model order reduction. *J. Sound Vib.* **502**, 116055 (2021).

[56] Lee, K. & Carlberg, K. T. Model reduction of dynamical systems on nonlinear manifolds using deep convolutional autoencoders. *J. Comput. Phys.* **404**, 108973 (2020).

[57] Simpson, T., Dervilis, N. & Chatzi, E. Machine learning approach to model order reduction of nonlinear systems via autoencoder and lstm networks. *J. Eng. Mech.* **147**, 04021061 (2021).

[58] Volkwein, S. Proper orthogonal decomposition: Theory and reduced-order modelling. *Lecture Notes*, University of Konstanz **4**(4), 1–29 (2013).

[59] Vlachas, K., Garland, A., Quin, D. D., Chatzi, E. Parametric reduced order modeling for component-oriented treatment and localized nonlinear feature inclusions. * Submitted to Nonlinear Dyn.*. (2023).

[60] Vlachas, K. *et al.* On the Coupling of Reduced Order Modeling with Substructuring of Structural Systems with Component Nonlinearities. In *Dynamic Substructures* Vol. 4 (eds Allen, M. S. *et al.*) (Springer, 2022).

[61] Allemang, R. J. The modal assurance criterion-twenty years of use and abuse. *Sound Vib.* **37**(8), 14–23 (2003).

[62] Vlachas, K. *et al.* Parametric Model Order Reduction for Localized Nonlinear Feature Inclusion. In *Advances in Nonlinear Dynamics* (eds Lacarbonara, W. *et al.*) 373–383 (Springer, 2022).

[63] Peherstorfer, B., Butnaru, D., Willcox, K. & Bungartz, H.-J. Localized discrete empirical interpolation method. *SIAM J. Sci. Comput.* **36**(1), A168–A192 (2014).

[64] Farhat, C., Chapman, T. & Avery, P. Structure-preserving, stability, and accuracy properties of the energy-conserving sampling and weighting method for the hyper reduction of nonlinear finite element dynamic models. *Int. J. Numer. Meth. Eng.* **102**(5), 1077–1110 (2015).

[65] Peherstorfer, B. Model reduction for transport-dominated problems via online adaptive bases and adaptive sampling. *SIAM J. Sci. Comput.* **42**(5), A2803–A2836 (2020).

[66] Peherstorfer, B. & Willcox, K. Online adaptive model reduction for nonlinear systems via low-rank updates. *SIAM J. Sci. Comput.* **37**(4), A2123–A2150 (2015).

[67] Farhat, C., Avery, P., Chapman, T. & Cortial, J. Dimensional reduction of nonlinear finite element dynamic models with finite rotations and energy-based mesh sampling and weighting for computational efficiency. *Int. J. Numer. Meth. Eng.* **98**(9), 625–662 (2014).

[68] Bishop, C. M. Latent Variable Models. In *Learning in Graphical Models* Vol. 89 (ed. Jordan, M. I.) 371–403 (Springer, 1998).

[69] Simpson, T., Tsialiamanis, G., Dervilis, N., Worden, K. & Chatzi, E. On the use of variational autoencoders for nonlinear modal analysis. In * IMAC XL*, 2022. Conference Location: Orlando, FL, USA; Conference Date: February 7-10, (2022).

[70] Vlachas, P. R., Arampatzis, G., Uhler, C. & Koumoutsakos, P. Multiscale simulations of complex systems by learning their effective dynamics. *Nat. Mach. Intell.* **4**(4), 359–366 (2022).

[71] Vlachas, P. R., Zavadlav, J., Praprotnik, M. & Koumoutsakos, P. Accelerated simulations of molecular systems through learning of effective dynamics. *J. Chem. Theory Comput.* **18**(1), 538–549 (2022). 34890204 10.1021/acs.jctc.1c00809

[72] Doersch, C. Tutorial on variational autoencoders. arXiv:1606.05908 (2016).

[73] Bengio, Y. *et al.* Scaling learning algorithms towards AI. *Large-Scale Kernel Mach.* **34**(5), 1–41 (2007).

[74] Kingma, D. P. & Ba, J. Adam: A method for stochastic optimization. In * 3rd International Conference on Learning Representations, ICLR 2015 - Conference Track Proceedings*, San Diego, California, ICLR. (2015).

[75] Srivastava, N., Hinton, G., Krizhevsky, A., Sutskever, I. & Salakhutdinov, R. Dropout: A simple way to prevent neural networks from overfitting. *J. Mach. Learn. Res.* **15**(56), 1929–1958 (2014).

[76] Vlachas, K., Agathos, K., Tatsis, K. E., Brink, A. R., & Chatzi, E. Two-story frame with bouc-wen hysteretic links as a multi-degree of freedom nonlinear response simulator. In * 5th Workshop on Nonlinear System Identification Benchmarks (2021)*, 6 (2021).

[77] Jonkman, J., Butterfield, S., Musial, W. & Scott, G. Definition of a 5-MW Reference Wind Turbine for Offshore System Development. Technical report, National Renewable Energy Laboratory, Golden, Colorado USA. (2009).

[78] Bathe, K. J. *Finite element procedures* (Klaus-Jurgen Bathe, 2006).

[79] Ismail, M., Ikhouane, F. & Rodellar, J. The hysteresis Bouc-Wen model, a survey. *Arch. Comput. Methods Eng.* **16**(2), 161–188 (2009).

[80] Vlachas, K., Tatsis, K., Agathos, K., Brink, A. R. & Chatzi, E. Two-story frame with bouc-wen hysteretic links as a multidegree of freedom nonlinear response simulator. Technical report, ETH Zurich. (2021).

[81] Ikhouane, F. & Rodellar, J. *Systems with Hysteresis: Analysis, Identification, and Control Using the Bouc-Wen Model* (Wiley, 2007).

[82] Chatzi, E. N. & Smyth, A. W. The unscented Kalman filter and particle filter methods for nonlinear structural system identification with non-collocated heterogeneous sensing. *Struct. Control. Health Monit.* **16**(1), 99–123 (2009).

