# Supplementary material for: VpROM: a novel variational autoencoder-boosted reduced order model for the treatment of parametric dependencies in nonlinear systems
Source: Sci Rep. 2024 Mar 13;14:6091. doi: 10.1038/s41598-024-56118-x (PMC11636824; doi:10.1038/s41598-024-56118-x)
Supplement: Supplementary file 1 — Supplementary Information. [file 41598_2024_56118_MOESM1_ESM.pdf]

Appendix A. VAE Architectures used

	Type	Weights	Activation	Dropout
**Encoder**	Dense	(200,48)	*tanh*	*None*
	Dense	(48,48)	*tanh*	*None*
	Dense	(48,48)	*tanh*	*None*
	Dense	(48,10)	*linear*	*None*
**Decoder**	Dense	(10,48)	*tanh*	*None*
	Dense	(48,48)	*tanh*	*None*
	Dense	(48,48)	*tanh*	*None*
	Dense	(48,200)	*linear*	*None*

Table A.1: Architecture of VAE used for hysteretic frame test case.

	Type	Weights	Activation	Dropout
**Encoder**	Dense	(32,18)	*tanh*	*None*
	Dense	(18,18)	*tanh*	*None*
	Dense	(18,18)	*tanh*	*None*
	Dense	(18,12)	*linear*	*None*
**Decoder**	Dense	(12,18)	*tanh*	*None*
	Dense	(18,18)	*tanh*	*None*
	Dense	(18,18)	*tanh*	*None*
	Dense	(18,32)	*linear*	*None*

Table A.2: Architecture of VAE used for wind turbine monopile case.
